# Acute bronchiolitis in infants, a review

**DOI:** 10.1186/1757-7241-22-23

**Published:** 2014-04-03

**Authors:** Knut Øymar, Håvard Ove Skjerven, Ingvild Bruun Mikalsen

**Affiliations:** 1 Department of Paediatrics, Stavanger University Hospital, PO Box 8100, N-4068 Stavanger, Norway; 2 Department of Clinical Science, University of Bergen, Bergen, Norway; 3 Institute of Clinical Medicine, University of Oslo, Oslo, Norway; 4 Department of Paediatrics, Oslo University Hospital, Oslo, Norway

**Keywords:** Bronchiolitis, Infant, Treatment

## Abstract

Acute viral bronchiolitis is one of the most common medical emergency situations in infancy, and physicians caring for acutely ill children will regularly be faced with this condition. In this article we present a summary of the epidemiology, pathophysiology and diagnosis, and focus on guidelines for the treatment of bronchiolitis in infants. The cornerstones of the management of viral bronchiolitis are the administration of oxygen and appropriate fluid therapy, and overall a “minimal handling approach” is recommended. Inhaled adrenaline is commonly used in some countries, but the evidences are sparse. Recently, inhalation with hypertonic saline has been suggested as an optional treatment. When medical treatment fails to stabilize the infants, non-invasive and invasive ventilation may be necessary to prevent and support respiratory failure. It is important that relevant treatment algorithms exist, applicable to all levels of the treatment chain and reflecting local considerations and circumstances.

## Introduction

Bronchiolitis is an acute lower respiratory tract infection in early childhood caused by different viruses, with coughing, wheeze and poor nutrition as the major symptoms [1-3]. A substantial proportion of children will experience at least one episode with bronchiolitis, and as much as 2-3% of all children will be hospitalized with bronchiolitis during their first year of life [1-4]. Bronchiolitis is the most common reason for hospitalization of children in many countries, challenging both economy, area and staffing in paediatric departments. Respiratory syncytial virus (RSV) is the most common virus causing bronchiolitis, occurring in epidemics during winter months [1,2].

Some infants, particularly those with risk factors, will have a severe course of bronchiolitis. Bronchiolitis is the most common medical reason for admission of children to intensive care units (ICU), providing challenges regarding ventilation, fluid balance and general support [5]. This may be a particular challenge for ICUs without a specialised paediatric section.

The aim of this article is to review current knowledge of severe bronchiolitis in infancy, with emphasis on the management.

## Methods

We performed a non-systematic search in PubMed up to January 2014, with the following words in different combinations; bronchiolitis, infants, children, severe, epidemiology, pathophysiology, guidelines, treatment, management, oxygen, hypertonic, saline, adrenaline, steroids, fluid, nutrition, continuous positive airway pressure (CPAP), bi-level positive airway pressure (BiPAP), high flow nasal cannulae and ventilation. Included studies and papers were not systematically evaluated regarding design and quality. However, we have emphasised recent guidelines, Cochrane reviews and other expert reviews.

### Clinical definition

There is no uniform definition of bronchiolitis, and no definite age limitation. In 2006, a subcommittee of the American Academy of Pediatrics (AAP) together with the European Respiratory Society (ERS) underlined that bronchiolitis is a clinical diagnosis, recognized as “a constellation of clinical symptoms and signs including a viral upper respiratory prodrome followed by increased respiratory effort and wheezing in children less than 2 years of age” [3]. In Europe, wheezing is regarded as a less important finding [2,6,7]. During recent years, several studies from Europe and the USA have included children only up to 12 months of age [2,8,9]. Children hospitalized for wheezing between 12 and 24 months of age may have a higher risk for having asthma, and with different pathophysiology and prognosis [1,2,6,10,11]. In this manuscript we will mainly focus on data from studies in infants with bronchiolitis younger than 12 months of age.

### Epidemiology

Approximately 20% of children develop bronchiolitis during their first year of life, and studies from the USA have found increasing rates of bronchiolitis (188/1000 infants in 1996/97 to 265/1000 in 2002/03) in this age group [6,12]. In a Norwegian study, the mean annual hospitalization incidence for RSV bronchiolitis was 21.7 per 1000 for children below 12 months [13], and in a large study from England the admission rate for all infants with bronchiolitis below 12 months of age was 24.2 per 1000 [14].

Bronchiolitis is generally seasonal, appearing most frequently in epidemics during the winter months [15]. For RSV, the same seasonal pattern is observed throughout the world, with most cases occurring from October until May on the northern hemisphere [15,16]. Adults with chronic obstructive lung disease and other immunocompromised patients may have RSV infection throughout the year and represent a reservoir of the virus [17,18].

Bronchiolitis is a disease with high morbidity, but low mortality. Death from respiratory failure in bronchiolitis is rare and range for RSV bronchiolitis from 2.9 (UK) to 5.3 (USA) deaths per 100 000 children below 12 months [19,20]. Differences may be caused by diagnostic procedures as well by socioeconomic conditions. A study from the UK underlines that the mortality rate for bronchiolitis in children below 12 months is low and falling, from 21.5 to 1.8 per 100 000 children (age 1 to 12 months) from 1979 to 2000, reflecting improvements in paediatric intensive care [21].

### Pathophysiology

RSV is the most common virus involved in children with bronchiolitis. In most studies it accounts for 60–80% of the bronchiolitis cases in children below 12 months of age [1,11,22-24]. In children below 12 months of age, Rhinovirus (RV) is the second most common virus (14–30%), thereafter human bocavirus (14–15%), human metapneumovirus (3-12%), entero-, adeno-, corona and influenza viruses (1–8%). Dual infections are reported in 20–30% of children, but does not seem to be associated with increased severity [6,11,22,25].

The infection starts in the upper respiratory tract, spreading to the lower airways within few days. The inflammation in the bronchioles is characterized by a peribronchial infiltration of white blood cell types, mostly mononuclear cells, and oedema of the submucosa and adventitia [2,6]. Damage may occur by a direct viral injury to the respiratory airway epithelium, or indirectly by activating immune responses [6].

Oedema, mucus secretion, and damage of airway epithelium with necrosis may cause partial or total airflow obstruction, distal air trapping, atelectasis and a ventilation perfusion mismatch leading to hypoxemia and increased work of breathing [1,2]. Smooth-muscle constriction seems to play a minor role in the pathologic process of bronchiolitis [2].

### Clinical characteristics

Bronchiolitis often starts with rhinorrhoea and fever, thereafter gradually increasing with signs of a lower respiratory tract infection including tachypnoea, wheezing and cough. Very young children, particularly those with a history of prematurity, may appear with apnoea as their major symptom [2,6]. Feeding problems are common.

On clinical examination, the major finding in the youngest children may be fine inspiratory crackles on auscultation, whereas high-pitched expiratory wheeze may be prominent in older children [2]. By observation, the infants may have increased respiratory rate, chest movements, prolonged expiration, recessions, use of accessory muscles, cyanosis and decreased general condition.

No formal scoring system for the severity of bronchiolitis exists, but a suggestion for the grading into mild, moderate and severe bronchiolitis based on guidelines from New Zealand and Scotland is given in Table 1[7,26].

**Table 1:** Assessment of the severity of bronchiolitis in infants < 12 months*

	**Mild bronchiolitis**	**Moderate bronchiolitis**	Severe bronchiolitis**
**Feeding**	Normal	Less than usual	Not interested
		>half the normal	< half the normal
**Respiratory rate**	< 2 months > 60/min	>60/min	>70/min
	> 2 months > 50/min		
**Chest wall recessions**	Mild	Moderate	Severe
**Nasal flare or grunting**	Absent	Absent	Present
**Sp02**	>92%	88-92%	<88%
**General behaviour**	Normal	Irritable	Lethargic

*Modified from New Zealand guidelines and SIGN guidelines [7,26].

**Not all criteria need to be met to categorize as severe bronchiolitis.

In a study including children with bronchiolitis from an out-patient clinic, the resolution of symptoms took more than 14 days in 40% of the children, and approximately 10% had symptoms after 4 weeks [6]. The median length of hospitalization in a large study including children below 12 months was only one day (IQR 0–3) [14], and in a Norwegian study the mean length of hospitalization was 80 hours (SD 67) [23].

Risk factors for bronchiolitis are male gender, a history of prematurity, young age, being born in relation to the RSV season, pre-existing disease such as bronchopulmonary dysplasia, underlying chronic lung disease, neuromuscular disease, congenital heart disease, exposure to environmental tobacco smoke, high parity, young maternal age, short duration/no breastfeeding, maternal asthma and poor socioeconomic factors. However, the majority of children hospitalized for bronchiolitis have no underlying condition [6,12,14].The same conditions may also be risk factors for a more severe course. Recently, specific gene polymorphisms have been associated with a risk for more severe bronchiolitis [27].

### Assessment

#### Clinical assessment

The diagnosis of bronchiolitis is made clinically, as described [3]. Risk factors for a severe course should be recognised, including young age which is associated with increased risk of apnoea, prolonged hospitalization, hypoxemia, admission to an ICU and the need of mechanical ventilation [2,3].

Pulse oxymetry should be included in the clinical assessment of bronchiolitis when possible, as it can detect hypoxemia not suspected by the clinical examination (Table 1) [2,3].

The course of bronchiolitis is variable, and repeated assessments should be performed, especially in infants with risk factors.

#### Laboratory assessment

Except for pulse oxymetry, no routine diagnostic tests have been shown to have a substantial impact on the clinical course of bronchiolitis, and recent guidelines and evidence-based reviews recommend that no diagnostic tests are used routinely [2,3,5,6,28]. Implementation of guidelines for the assessment and treatment of infants with bronchiolitis has reduced the use of diagnostic as well as therapeutic options, with a further reduction in costs and length of stay [2,29-33].

The clinical course and management of bronchiolitis are similar and not influenced by identification of the viral agent [2,3]. However, identifying a viral etiology is shown to reduce the use of antibiotics, the number of investigations and the length of stay [6,30]. Dependent on the setting, a viral diagnosis may be warranted for cohorting of patients and may reduce nosocomial infections, which may have impact on the long-term prognosis of the child [3]. However, this has been questioned by others [34].

Examination by chest X-ray may increase the rate of antibiotic prescription without improving any outcome, and may in less than 1% reveal lobar consolidations suggesting the need of antibiotics [5,35]. An X-ray may, however, be more likely to add positively in children with high and prolonged fever, oxygen saturation < 90%, chronic cardiopulmonary disease and in children in need of admission to an ICU or mechanical ventilation [5,36].

Blood tests are commonly taken in children with bronchiolitis, but are not of clinical value in most patients and not recommended routinely [2,3,37]. Tests to be included may be total blood count and C-reactive protein if a secondary bacterial infection is suspected, and electrolytes in infants with feeding problems and signs of dehydration. Blood gases is warranted and useful in infants with severe respiratory distress and potential respiratory failure [6].

### Management

#### General management

Management of acute bronchiolitis is generally supportive, as no medical treatment has shown to improve important clinical outcomes, such as length of hospital stay, use of supportive care or transfer to an intensive care unit. A conservative, “minimal handling” approach seems beneficial, especially for the youngest age group (<3 months) [1,2,23]. A prone position may improve oxygenation and is suggested for infants if they are carefully observed [1,38]. Careful nasal suctioning may be beneficial in infants with copious secretion [1,39].

#### Oxygen

Oxygen should be administered in hypoxic infants with bronchiolitis, and administered via nasal cannulae or a face mask [1]. However, there is no consensus on what level of oxygen saturation (SpO_2_) oxygen support should be aiming at, and no randomized controlled trials have compared alternative oxygen supplementation regimes [1,40]. In the UK, oxygen is commonly given to achieve a SpO_2_ of 92-95%, while the AAP recommends a limit of SpO2 of 90% in otherwise healthy children [1-3,7,39]. Observational studies, however, indicate that a goal of 90%, as compared to 94%, has the potential to significantly reduce length of hospital stay [41,42], and the AAP guidelines recommend a reduced level of monitoring as the infants improve [3].

#### Fluid and nutrition

Maintaining hydration is an important part of the care of infants with bronchiolitis. The respiratory distress due to increased work of breathing may cause inadequate feeding and eventually lead to poor hydration [1]. Further, tachypnoe and fever increases fluid loss, potentially worsening the dehydration [43,44]. Oral feeding may be sustained in milder cases, if needed by small volume frequent feed, and breastfeeding should be encouraged. However, a substantial part of infants hospitalized for bronchiolitis will be in need of fluid supplementation, either as intravenous (IV) fluid or with enteral feeding by gastric tube (GT) [1,2,44]. Traditionally, IV fluid has been given in many countries, and is also recommended in the present AAP guidelines [3]. The advantage of IV fluids could be a decreased risk of aspiration and no interference with breathing [45,46], but with the disadvantage of possibly creating a catabolic state due to low calorie intake, and bearing a higher risk of fluid overload and electrolyte imbalance [43,44,46]. Through GT feeding, infants may achieve a better nutritional status and nitrogen balance, which may be beneficial for recovery, and may be a route for giving expressed breast milk [44,47]. Feeding by GT may be given as boluses, or continuously in case of major respiratory distress [1].

Currently there is not sufficient evidence for or against the use of GT feeding in infants with bronchiolitis [46], and in a recent large study from Australia no differences in major outcomes were found between the two methods [48]. However, feeding by GT has been increasingly adopted, and used as routine in some countries [41,49,50], including the recent guidelines of the Norwegian Paediatric society [51]. In a large Scottish study of bronchiolitis, no children received IV fluids, and no complications related to feeding by GT were reported [41]. Recently, a minor randomized pilot study comparing IV fluid and feeding of GT showed no difference regarding the duration of oxygen supplementation or length of stay between the two methods [43].

Few studies have addressed the appropriate amount of fluid to be given during replacement in bronchiolitis. Guidelines recommend that infants should receive enough fluid to restore fluid loss and avoid dehydration, and the amount should not exceed 100% of daily fluid requirements, normally set to 100 ml/kg for infants < 10 kg [3]. However, fluid retention due to inappropriate secretion of antidiuretic hormone has been reported in bronchiolitis, and clinicians should be aware of the possibility of overhydration [1,52,53]. Consequently, 70-80% of the daily requirements may be recommended, especially in those with severe disease [1,3,44]. In these children, close monitoring of weight, serum and urine-osmolality and serum electrolytes may guide treatment [1]. Probably, possible overhydration will be a less problem during enteral feeding, permitting the body to absorb the needed amount of fluid and electrolytes.

#### Inhaled saline

Inhaled normal saline (0.9%) is commonly used for children with bronchiolitis to increase clearing of mucous, and is included as placebo in many studies evaluating the effect of bronchodilators or hypertonic saline. However, we are not aware of any randomised study comparing normal saline with no treatment, and normal saline is not suggested in current guidelines and reviews [1-3,6,39]. Consequently, no recommendations can be given.

Inhaled hypertonic saline has, in patients with various diseases, shown to increase mucociliary clearance possibly through induction of an osmotic flow of water to the mucus layer and by breaking ionic bonds within the mucus gel [54]. Recent metaanalyses including more than 1000 infants with mild to moderate bronchiolitis, concluded that the use of hypertonic saline (3-5%) may reduce the length of hospital stay and the rate of hospitalization [55,56]. However, due to the possible side effect of bronchospasm, all but few patients received a combination with a bronchodilator. The optimal delivery interval, concentration and delivery device remain unclear. The short term effect was conflicting, as four trials showed no such effect [55]. In a recent study, 7% hypertonic saline with epinephrine did not have any effect on the clinical severity score [57].

A recommendation of hypertonic saline inhalations based on the current evidence must include a bronchodilator. As recent evidence strongly supports the “minimal handling” approach to infants with bronchiolitis [23], we do not support such a recommendation at this time. Several trials with hypertonic saline without bronchodilators are ongoing, from which results may adjust guidelines [1].

#### Inhalations with bronchodilators

In addition to bronchodilation, inhalation with adrenaline may reduce mucosal swelling, which has led to frequent use in infants with bronchiolitis. However, a clinically important, significant effect has been documented for neither adrenaline nor beta-2-agonists. Studies on short-term effects show conflicting results. A recent Cochrane review concludes that inhaled (racemic) adrenaline does not improve important clinical outcomes such as length of hospital stay or the use of supportive care in moderate to severe bronchiolitis inpatients [58]. This is supported by a recent large Norwegian randomised controlled trial (RCT) of 404 infants [23]. In this study, treatment “as needed” rather than on a fixed schedule resulted in less inhalations (12 vs. 17 per day), shorter hospital stay (47.6 vs. 61.3 hours), less use of supplemental oxygen (38.3 vs. 48.7%) and less ventilatory support (4.0 vs. 10.8%). The effect was predominantly seen in children <3 months (25 hours reduced hospital stay), which also tended to have a negative effect of adrenaline compared to saline, supporting a conservative approach particularly in this age group. Adrenaline is therefore not recommended as a standard treatment in infants with bronchiolitis, but a trial might be performed in children >3 months, with critical evaluation of effect with respect to continuation of administration [23]. Beta-2-agonists are not recommended for infants with bronchiolitis [59,60].

#### Steroids

A recent metaanalysis including 17 RCTs concluded that there is no beneficial effect of systemic corticosteroids in children with bronchiolitis, neither on rate of hospitalization for outpatients nor on length of stay for inpatients [61]. However, one study has shown a beneficial effect of dexamethasone (0.15 mg/kg every 6 h for 48 h) in mechanically ventilated children, suggesting that this may be an option in critically ill patients [62]. Further, the combined therapy with inhaled epinephrine and high-dose oral dexamethasone (1 mg/kg at presentation and 0.6 mg/kg for an additional 5 days) appeared to reduce the rate of hospital admission in a small study [63]. However, this treatment cannot be recommended until evaluation in larger studies has been done.

#### Additional medication

Antibiotics is commonly used in children with lower respiratory tract infections, but a Cochrane review including 543 infants concludes that there is no evidence for the use of antibiotics in general [64]. However, antibiotics may more frequently be warranted due to concurrent bacterial infections in infants with severe disease, especially those needing mechanical ventilation [65]. There is no role for antiviral therapy in bronchiolitis [66].

Surfactant therapy has been suggested for critically ill patients on mechanical ventilation. So far, this has been evaluated in only three small studies, and a recent Cochrane review concluded that there is insufficient evidence for such treatment [67]. The use of recombinant human deoxyribonuclease has not been efficacious on any of the outcome variables in children with bronchiolitis [68].

#### Non-invasive and invasive ventilation

Continuous positive airway pressure (CPAP) with a nasal tube or a nasal mask has been widely used in children with moderate or severe bronchiolitis. CPAP may act by recruiting collapsed airways and the corresponding alveoli, giving a reduction in mean airway resistance. This further increases the emptying of the lungs during expiration, resulting in a decreased hyperinflation and work of breathing, and improved gas exchange [69,70].

Contrasting the widely use, the documentation for the use of CPAP in bronchiolitis is sparse. A recent systematic review concluded that the evidence supporting the use of CPAP to reduce PCO_2_ and respiratory distress is of low quality, and it has not been shown that the use of CPAP reduces the need for invasive ventilation [69]. Only two small RCTs have been performed [71,72], the other studies have a before-after design [73-77]. However, these studies have found that the use of CPAP in bronchiolitis is safe, and on average reduces the capillary PCO_2_ from before to shortly after CPAP is initiated with 0.8 to 1.3 kPa [69].

The pressure used during ventilation with CPAP is commonly set to 4–8 cm H_2_O, and a pressure of 5 cm H_2_O has been efficient in reducing PCO_2_. Recently, a prospective study suggested that a nasal CPAP level of 7 cm H_2_O was most efficient in reducing respiratory distress and improving the breathing pattern [78].

Heliox is a mixture of helium and oxygen and a low-density gas. It may have a beneficial role in bronchiolitis by transforming turbulent into laminar gas flow and thereby improving oxygenation and the washout of CO_2_[79]. The combination of heliox and CPAP (CPAP-He) has been evaluated in three studies. All the studies included few children, one quasi RCT and two before-after studies [69,80-82]. All three studies showed a significantly decrease in transcutaneous or arterial PCO_2_ and respiratory distress. However, as no blinded RCT has been performed, it must be concluded that more evidence is needed before CPAP-He can be included in guidelines [69]. Heliox therapy without the use of a tight CPAP or combined with a nasal cannulae has been shown to be ineffective [83].

Though no uniform criteria have been published, common criteria for which children should be treated with CPAP are respiratory distress, high oxygen requirement or increasing pCO_2_ and apnoeas [1]. In a recent study, the strongest predictors for CPAP treatment were oxygen requirement, low oxygen saturation, younger age and higher respiratory rate [84].

The use of heated humidified high-flow nasal cannulae (HFNC) has increasingly been introduced as an alternative to nasal CPAP [85-91], also in a general paediatric ward [92]. The method is currently used in neonatal medicine [93], and may generally act by increasing the pharyngeal pressure, leading to a reduction in respiratory efforts and improving respiratory distress [94]. Based on the current literature, a recent review concludes that HFNC may be feasible in infants with bronchiolitis and may decrease the need for intubation [87,90]. HFNC may be better tolerated than nasal CPAP [86,89,95], and larger paediatric units have replaced CPAP with HFNC as the first-line non-ventilatory support in bronchiolitis [86]. However, no randomized trial has yet evaluated the effect in bronchiolitis patients, and the most recent study concludes that there is insufficient evidence to determine the effectiveness of HFNC in infants with bronchiolitis [96]. Serious air leak syndrome has been shown in some cases of children treated with HFNC [97].

The safety of HFNC and CPAP may be arguments for the early introduction of non-ventilatory support in children with moderate bronchiolitis [69]. However, mechanical ventilation may still be necessary in infants with insufficient support by nasal CPAP or HFNC. Risk factors include prematurity, low birth weight and bronchopulmonary dysplasia, and further those with apnoe, low oxygen saturation, poor oral intake and severe retractions on admission [98,99].

There is no consensus on which ventilator technique is the best for children with bronchiolitis [70,100]. Both volume and pressure cycled ventilation has been used, with a large variation in ventilator rates (10–60 beats per minute), maximum pressure (20–50 cm H_2_O) and tidal volume (6–20 ml/kg) [70]. The use of PEEP is also varying, from 0 to 15 cm H_2_O. The use of high frequency oscillation has been successful in some case reports [101]. However, it is suggested that infants with hyperinflation may benefit from slower rates and longer expiratory times [70].

For those very few not controlled on mechanical ventilation (in most cases associated with severe bronchopulmonary dysplasia), extracorporeal membrane oxygenation has been shown to have some benefit [70,98].

### Preventive measures

It is important to avoid nosocomial spread of RSV and other respiratory viruses from children with bronchiolitis [102]. RSV can survive up to seven hours on surfaces and is transmitted directly or indirectly by touch [103]. Further, air sampling in subjects infected with RSV has detected RSV RNA up to 700 cm from head of the patients bed [104]. Hand decontamination by antimicrobial soap or alcohol based hand rubs before and after patient contact, and also after removing gloves and after contact with possible decontaminated objects, is an important strategy for primary prevention [3]. However, wearing masks has not been shown to have additional benefit [3].

### Management plan

**Figure 1:**
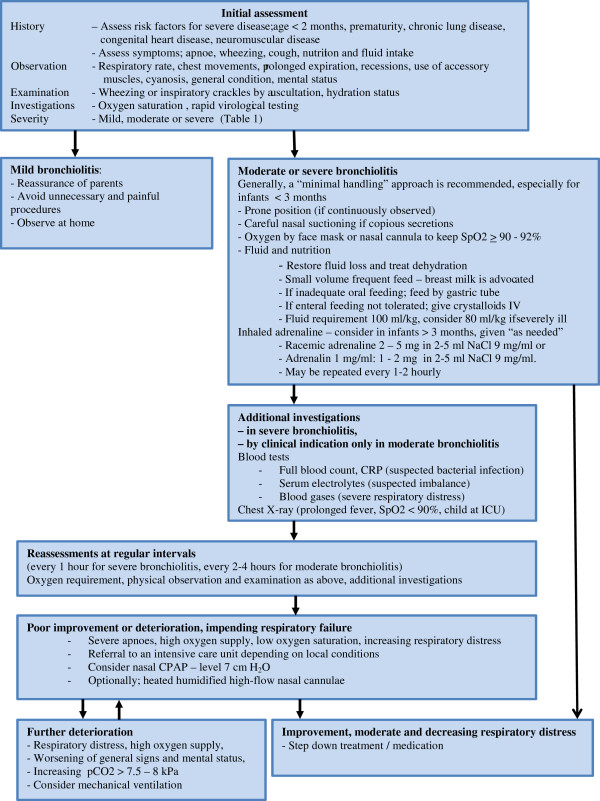
Treatment algorithm for infants with bronchiolitis.

Based on the above considerations and recent guidelines, we suggest a treatment plan for acute bronchiolitis in children, including dose recommendations (Figure 1).

All institutions caring for children with bronchiolitis should provide a clear in-house treatment algorithm to their staff, taking local considerations and circumstances into account.

### Differential diagnostic considerations

In most cases, the diagnosis of bronchiolitis is clinically evident and no further tests are indicated to rule out other diagnosis [2]. However, other diagnosis may be considered in a child with atypical presentations including severe respiratory distress, and recurrent symptoms, and in a child presenting with otherwise typical symptoms, but with no signs of a viral infection [2]. Differential diagnosis may include gastroesophageal reflux, laryngotracheobroncholamacia, pertussis, foreign body aspiration, vascular ring and other mediastinal obstructions or other congenital lung diseases [2,6]. Asthma may be considered in the oldest infants with recurrent episodes of wheeze, but the overlap with asthma is less likely when bronchiolitis is only defined in infants younger than 12 months of age [6].

### Outcome

Children hospitalized with bronchiolitis in infancy have an increased risk of subsequent asthma, reduced lung function and increased bronchial hyperresponsiveness [24,105]. Except from in the studies by Sigurs et al. [8], they do not seem to have an increased risk of atopy [106]. The increased risk of asthma is particularly found among children hospitalized with RSV negative bronchiolitis or bronchiolitis due to Rhinovirus [24,107], whereas the association between RSV bronchiolitis in infancy and subsequent respiratory morbidity decreases with age [108]. The association between bronchiolitis and later asthma is complex and probably related to viral etiology, genetic, structural, immunological, inflammatory and environmental mechanisms [109].

## Conclusion

Bronchiolitis is the most common reason for hospitalization during infancy, being a burden for the child and family, and bearing huge costs for the healthcare systems. The main principles for treatment include minimal handling, maintenance of oxygen saturation, fluid balance and nutrition. Other therapeutic options are inhalations with epinephrine, normal saline or hypertonic saline, but the evidences for their use are sparse. CPAP and heated humidified high-flow nasal cannulae are commonly used in those with respiratory failure, but more high-quality studies are needed to prove their efficacy. Very few children may be in need of mechanical ventilation.

## Competing interests

The authors declare that they have no competing interests.

## Authors’ contributions

KØ initiated and coordinated the writing of the manuscript. KØ, HOS, and IBM all contributed substantially to the literature search and writing, and read and approved the final manuscript.
